# Effects of *Saccharomyces cerevisiae* Hydrolysate on Broiler Performance and Gut Health

**DOI:** 10.3390/ani15172531

**Published:** 2025-08-28

**Authors:** Marcello Comi, Davide Lanzoni, Vera Perricone, Xian-Ren Jiang, Jing Lin, Hai-jun Zhang

**Affiliations:** 1 Department of Human Science and Quality of Life Promotion, Università Telematica San Raffaele, 00166 Roma, Italy; 2 Department of Veterinary Medicine and Animal Sciences (DIVAS), University of Milan, 26900 Milan, Italy; 3 Key Laboratory of Feed Biotechnology of Ministry of Agriculture and Rural Affairs, Institute of Feed Research, Chinese Academy of Agricultural Sciences, Beijing 100081, China

**Keywords:** broiler, hydrolysate, intestine, microbiota, performance, *Saccharomyces cerevisiae*, yeast

## Abstract

In recent years, scientific interest in functional ingredients capable of replacing the non-therapeutic use of antibiotics in animal feed has intensified, fostering the exploration of novel additives such as *Saccharomyces cerevisiae* hydrolysate (SCH). This study investigated the effect of dietary SCH supplementation on growth performance, intestinal morphology, local immune response, and cecal microbiota composition in Ross 308 broiler chickens. A total of 300 one-day-old male chicks were randomly assigned to two experimental groups, receiving either a standard diet or a diet supplemented with SCH (500 mg/kg during the starter and grower phases; 250 mg/kg during the finisher phase). SCH supplementation significantly improved growth performance during the finisher phase, with increases in final body weight (*p* = 0.025), average daily gain (*p* = 0.049), and average daily feed intake (*p* = 0.027), without significant changes in feed conversion ratio (*p* > 0.05). Favourable intestinal morphological modifications were observed, with a significant increase in villus height to crypt depth ratio in both the jejunum and ileum at days 28 (*p* = 0.035 and 0.002, respectively) and 42 (*p* < 0.001). The expression of pro-inflammatory cytokine genes was significantly reduced, with lower levels of TNF-α, IL-1β, and IL-6, while tight junction protein genes ZO-1 and Occludin were significantly upregulated (*p* < 0.05). Microbiota profiling revealed higher alpha diversity and greater abundance of *Prevotella*. These findings highlight SCH as a promising dietary strategy to improve broiler performance, intestinal function, and sustainability in poultry production systems.

## Simple Summary

The removal of antibiotic growth promoters from poultry production has increased interest in alternative feed additives that can support gut health and performance. This study evaluated the effects of dietary supplementation with *Saccharomyces cerevisiae* hydrolysate on growth performance, intestinal morphology, immune response, and gut microbial composition in broiler chickens. A total of 300 male broilers were assigned to diets with or without the yeast hydrolysate. Birds receiving the supplement showed improved body weight and feed intake during the finisher phase. Intestinal histology revealed increased villus height and improved villus-to-crypt ratios, indicating enhanced absorptive capacity. Gene expression analysis showed reduced markers of inflammation and increased expression of proteins associated with gut barrier integrity. Microbial analysis of the cecum indicated greater diversity and higher abundance of beneficial bacteria. These findings suggest that *Saccharomyces cerevisiae* hydrolysate can enhance growth performance and support intestinal health in broilers, offering a promising nutritional strategy for antibiotic-free poultry systems. Its use could contribute to more sustainable production while addressing consumer and regulatory concerns around antibiotic resistance.

## 1. Introduction

In recent decades, the systematic use of antibiotics as growth promoters in intensive livestock farming has contributed significantly to improving production efficiency, especially in the poultry sector. However, the non-therapeutic use of antibiotics has been progressively challenged due to the increasing spread of antibiotic resistance, now recognised as a major threat to global public health [1]. Consequently, increasingly strict regulations, have restricted or banned their use in farm animals, requiring the livestock sector to adopt alternative strategies that can support animal welfare, food safety and production performance in the absence of antibiotics as growth promoters.

In response to this need, research has focused on identifying new feed additives with functional effects, including prebiotics, probiotics, postbiotics, organic acids, plant extracts and microbial derivatives [2]. Amongst these, as reported by Lin et al. [3], yeast hydrolysate, and in particular that obtained from *Saccharomyces cerevisiae* (*S. cerevisiae* hydrolysate, SCH), has shown considerable potential due to its nutritional and bioactive composition. SCH is produced through autolysis processes or enzymatic hydrolysis of fermented yeast biomass, and contains a variety of functional components including free nucleotides, B-vitamins, essential amino acids, β-glucans and mannan-oligosaccharides derived from the cell wall [4].

Each of these elements has demonstrated specific activities relevant to maintaining intestinal health and modulating the immune response. Nucleotides, for instance, are involved in nucleic acid synthesis, cell turnover and regulation of immune functions; they also promote regeneration of the gastrointestinal mucosa, especially under stress conditions or during rapid growth phases [5,6]. β-glucans and mannan-oligosaccharides, on the other hand, play a role in modulating innate immunity and act as prebiotics, contributing positively to the composition of the gut microbiota [7]. In poultry, the provision of such compounds through hydrolyzed yeast has been shown to improve growth and laying performance [8,9], enhance immune responses to vaccination [10,11], and favour beneficial gut microbiota while limiting pathogens [12,13].

The gastrointestinal tract, with its complex microbial ecosystem, plays a central role in feed efficiency, protection against enteric pathogens, and the maturation of the immune system. Maintaining the integrity of the gut barrier and the balance of the caecal microbial community is an essential prerequisite for ensuring the overall health of the animal and, consequently, its production performance [14]. In this context, SCH has been associated with numerous beneficial effects, including improving nutrient digestibility [15], supporting intestinal function [16], increasing resistance to infection [17] and modulating inflammatory responses [18]. Although several studies have highlighted the efficacy of SCH in improving performance in broiler chickens [3,13,19], the biological mechanisms underlying these effects remain largely to be investigated. In particular, the potential role of SCH in regulating the gut barrier, mucosal immunity and the composition of the caecal microbiota in poultry needs further focused studies.

Therefore, the present study aimed to evaluate the effects of dietary supplementation with SCH on key variables related to health and productivity in broilers: growth performance, gut morphology, local immune response and tight junction protein expression. Subsequently, the composition of the caecal microbiota was analysed by high depth sequencing with the aim of identifying possible treatment-induced changes.

## 2. Materials and Methods

In the present study, a yeast hydrolysate obtained from *S. cerevisiae* (I-Care, batch number: 210210; production date: February 2021), supplied by Prosol S.p.A. (Madone, Italy), was used. The compositional analysis of the supplement showed a content of 38% crude protein, with bioactive fractions comprising 4.9% glutamic acid, 3.5% nucleotides, 23% β-glucans and 15% mannan-oligosaccharides.

### 2.1. Experimental Design and Management

The entire experimental cycle was conducted in a temperature-controlled environment, strictly following the recommended protocols for rearing Ross strain 308. At hatching, the chicks were kept at an initial temperature of 33 ± 0.5 °C, which was gradually reduced to 21.0 ± 1.0 °C at the end of the fourth week of life. The lighting schedule was 23 h light:1 h darkness for the first four days of the trail and then gradually reduced to 18 h light:6 h darkness by d 8. Animals were housed in four-level batteries; each experimental unit comprised 25 individuals and consisted of cages measuring 180 × 120 × 45 cm, with flooring covered by a plastic-coated grid (stocking density of 11.6 chicks/m^2^). A total of 300 one-day old male Ross 308 broiler chicks (BW: CTR = 41.35 ± 0.37 g and TRT = 41.28 ± 0.33 g), originating from a local hatchery, were randomly assigned to two experimental treatments, as described below. Each treatment comprised six replicates, each consisting of 25 animals. Each cage was equipped with one feeder and four drip troughs. During the entire trial period (total = 42 days), access to both feed and water was provided ad libitum. The health status of the subjects was checked daily, with any behavioural or clinical changes recorded and therapeutic interventions applied where necessary.

The experimental design involved the comparison of two experimental groups: (I) a control group (CTR), receiving a diet based on wheat and soybean meal, formulated according to the nutritional requirements set forth by the National Research Council [20] (Table 1); (II) a treated group (TRT), receiving the same diet supplemented with SCH. Specifically, SCH was supplemented at 500 mg/kg in the starter (days 1–14) and grower (days 15–28) phases, and 250 mg/kg in the finisher phase (days 29–42). The inclusion level was set at the dosage recommended by the manufacturer for commercial use, which was established based on results from previous internal trials. The three diets administered during the different phases were analysed to determine the content of dry matter (DM), crude protein (CP) and ether extract (EE), according to GB/T 6435 [21], GB/T 6432 [22] and GB/T 6433 [23], respectively (Table 2).

The trial was conducted in compliance with current animal welfare regulations, subject to approval by the Committee for Animal Care and Use of the Feed Research Institute, Chinese Academy of Agricultural Sciences, Beijing (protocol no. FRI-CAAS-20210903).

### 2.2. Performance Evaluation

Production performance was monitored on days 14, 28 and 42 by measuring body weight (BW) and feed intake (FI). From these data, average daily gain (ADG), average daily feed intake (ADFI) and feed conversion ratio (FCR) were calculated. Mortality was recorded daily and used to correct the FCR.

### 2.3. Tissue Collection and Morphological, Immunological and Molecular Evaluation of the Intestine

At days 14, 28 and 42, one subject per replicate, with a body weight within ±5% of the mean, was selected, marked, starved overnight, weighed and then euthanised; intestinal tissues were collected to analyse intestinal relative index (IRI) and morphology. The IRI was calculated for each tract (duodenum, jejunum, ileum and cecum) by relating the weight of the intestinal tract to the body weight according to the formula:

$$IRI=\left[intestinal\;segment\;empty\;weight\left(g\right)÷\left.body\;weight\;(g)\right]\times 100\right.$$

Subsequently, central segments of the duodenum, jejunum, and ileum were aseptically dissected, washed with sterile 0.9% saline, fixed in 10% buffered formalin and stored at 4 °C for morphological analysis.

Samples were stained with toluidine blue and analysed under a light microscope to measure villus height (VH), crypt depth (CD) and villus/crypt ratio (VCR).

On days 28 and 42, mucosal samples of the jejunum and ileum were collected from the same six animals per group used for intestinal morphological evaluation. Secretory immunoglobulin A (sIgA) levels were quantified by using the immunohistochemical assay described by Gao et al. [24]. In parallel, mRNA extracted from the jejunal mucosa was used for gene expression analysis of the pro-inflammatory cytokines (TNF-α, IL-1β, IL-6, and IFN-γ) and tight junction proteins (Claudin-1, Occludin and Zonula Occludens-1-ZO-1), by quantitative RT-PCR (Table 2). β-actin was used as a housekeeping gene for the normalisation of expression data. Total RNA of jejunum mucosa samples was extracted by Trizol reagent (Thermo Fisher Scientific, Wilmington, DE, USA) and determined the purity and concentration by NanoDrop 2000 spectrophotometer (Thermo Fisher Scientific, Wilmington, DE, USA). Then, 1 μg RNA of each sample was used to reverse-transcribe into cDNA using TransScript First-Strand cDNA Synthesis SuperMix (TransGen Biotech, Beijing, China) following the manufacturer’s guidelines. The PowrUp SYBR Master Mix (Thermo Scientific, Thermo Fisher Scientific, Wilmington, DE, USA) was used to carry out real-time quantitative polymerase chain reaction (qRT-PCR) on a QuantStudio^®^5 real-time PCR Design & Analysis system (Applied Biosystems, Foster City, CA, USA). Each sample was measured in duplicate. Primers sequences used in this study was shown in Table 3. The relative mRNA expression levels were normalised to avian β-actin by the 2^−ΔΔCt^ method [25].

### 2.4. Profile of the Ceca Microbiota

On day 42, caecal contents were collected from the same six subjects per treatment used for morphological and biochemical evaluation for gut microbiota analysis. Microbial DNA was extracted from 300 mg of content using the E.Z.N.A.^®^ Soil DNA kit (Omega Bio-tek, Norcross, GA, USA). The V3-V4 hypervariable region of the 16S rRNA gene was amplified by PCR with primers 338F (5′-ACTCCTACGGAGCAG-3′) and 806R (5′-GGACTACHVGGTWTCTAAT-3′), following standard conditions: initial denaturation at 95 °C for 2 min, 25 cycles of 95 °C for 30 s, 55 °C for 30 s, 72 °C for 30 s, and a final extension at 72 °C for 5 min. The amplified products were purified and sequenced in paired-end (2 × 250 bp) using Illumina MiSeq platform at Shanghai Majorbio Bio-Pharm Technology Co., Ltd. (Shanghai, China). The raw sequences were deposited in the NCBI Sequence Read Archive database.

### 2.5. Statistical Analysis

The statistical evaluation of growth performance, intestinal relative index, morphometric parameters, and gene expression levels was conducted using SAS software (version 9.2; SAS Institute Inc., Cary, NC, USA). Each battery was treated as the experimental unit for performance-related variables, while individual birds represented the experimental unit for intestinal measurements. A one-way analysis of variance (ANOVA) was applied to detect significant differences among treatment groups. Duncan’s multiple range test was used for post hoc comparisons. Results were expressed as means ± standard deviation (SD), and significance was set at *p* < 0.05.

Regarding the microbial community analysis, raw paired-end reads obtained from sequencing were demultiplexed and subjected to quality control using the QIIME pipeline (version 1.17; [26]). Sequences with lengths shorter than 150 base pairs or with average Phred scores below 20 were discarded [27]. High-quality sequences were clustered into operational taxonomic units (OTUs) based on 97% sequence identity. OTUs were taxonomically annotated using the Greengenes database. Alfa-diversity metrics and rarefaction curves were calculated through QIIME [28], while Beta-diversity was assessed using principal coordinate analysis (PCoA) and partial least squares discriminant analysis (PLS-DA).

## 3. Results

### 3.1. Performance Evaluation

Values for the evaluation of production performance are reported in Table 4. As observed, supplementation with SCH did not significantly influence broiler performance during the starter (1–14 days) and grower (15–28 days) phases, with BW, ADG, ADFI and FCR being similar between groups. However, in the finisher phase (29–42 days), supplementation with SCH resulted in a significant improvement in production performance, with an increase in final BW (*p* = 0.025), ADG (*p* = 0.049) and ADFI (*p* = 0.027), compared to the control group. Over the entire experimental period (1–42 days), TRT broilers showed significantly higher ADG and ADFI (*p* = 0.015 and *p* = 0.034, respectively) compared to CTR, without significant changes in FCR or mortality.

### 3.2. Morphological, Immunological and Molecular Evaluation of the Intestine

As shown in Table 5, IRI showed no significant differences between TRT and CTR in almost all intestinal districts and at the different sampling times (14, 28 and 42 days). The only exception was observed at 28 days in the jejunum tract, where broilers fed a diet containing SCH showed a higher IRI than the control group (*p* = 0.021).

With regard to intestinal morphology (Table 6), at day 14 there were no statistically significant differences between the groups in the analysed parameters (VH, CD and VCR) —in any of the intestinal tracts considered. However, at day 28, favourable morphological changes were observed in TRT. In particular, in the jejunum, the VCR was significantly increased compared to the control (*p* = 0.035). In the ileum, there was a significant increase in VH (*p* = 0.013), a reduction in CD (*p* = 0.032) and a subsequent increase in VCR *(p =* 0.002). Finally, at day 42, although no significant differences in VH and CD were found between groups, VCR was significantly higher in TRT compared to CTR, both in jejunum (*p* < 0.001) and ileum (*p* < 0.001).

As shown in Table 7, the concentration of sIgA was not significantly affected by SCH supplementation, neither at 28 nor at 42 days, in both jejunum and ileum.

However, gene expression analysis of pro-inflammatory cytokines (Table 8) showed a more pronounced immunomodulatory effect with advancing age. In particular, a significant reduction in IL-1β expression was observed at 28 days (*p* = 0.018). This effect was accentuated at 42 days, when supplementation resulted in a significant reduction in the expression of TNF-α (*p* < 0.001), IL-1β (*p* = 0.002) and IL-6 (*p* = 0.049).

In line with the observed effects on the modulation of the immune response, supplementation with SCH also exerted a significant impact on the integrity of the intestinal barrier (Table 9). In particular, gene expression of ZO-1 and Occludin was significantly increased in TRT compared to CTR, both at 28 and 42 days (ZO-1 at 28 and 42 d: *p* < 0.001; Occludin: at 28 d *p* = 0.005 and 42 d *p* < 0.001). With regard to Claudin-1, although a numerical increase in expression was detected in TRT, the difference did not reach statistical significance, however, suggesting a possible trend towards an overall strengthening of the tight junction structure.

### 3.3. Evaluation of Caecal Microbiota

After filtering, an average of 52,912 reads per sample was obtained. Sequencing depth was assessed by plotting rarefaction curves for OTU richness, and most samples reached plateaus, indicating that the sequencing depth was sufficient to capture the majority of microbial diversity. Across all samples, the control group contained 780 OTUs, while the treatment group contained 622 OTUs, with 397 OTUs shared between the two groups.

**Figure 1:**
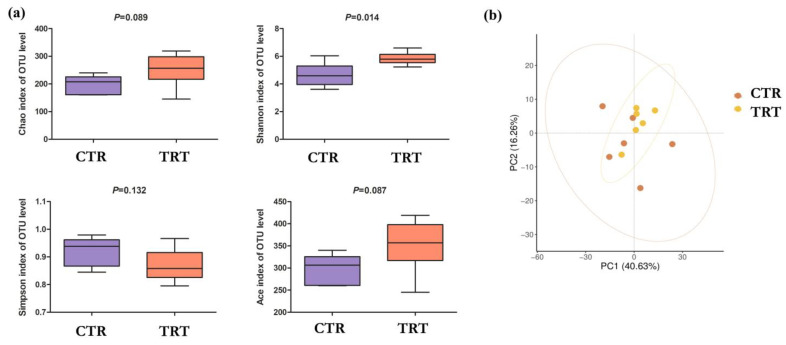
Effects of *S. cerevisiae* hydrolysate on the alfa (**a**) and beta (**b**) diversity of the caecal microbiota in broilers. OTU: operational taxonomic units. CTR: control group; TRT: treated group with *S. cerevisiae* hydrolysate. (n = 6 replicates (subjects) per group).

The alfa diversity analysis (Figure 1a) showed a positive effect of SCH supplementation on the intestinal microbial composition in broilers. In particular, the Shannon index was significantly higher in TRT than in CTR (*p* = 0.014), indicating a greater diversity and uniformity of the bacterial community. Although the Chao (*p* = 0.089), ACE (*p* = 0.087) and Simpson (*p* = 0.132) indices also showed an increasing trend in the SCH group, these differences did not reach statistical significance.

With regard to beta diversity (Figure 1b), PCoA showed no significant differences in the overall microbial composition between the two groups. The samples belonging to CTR and TRT were superimposable in the principal component space (PC1: 40.63%; PC2: 16.26%), suggesting that supplementation did not profoundly alter the overall structure of the gut bacterial community.

**Figure 2:**
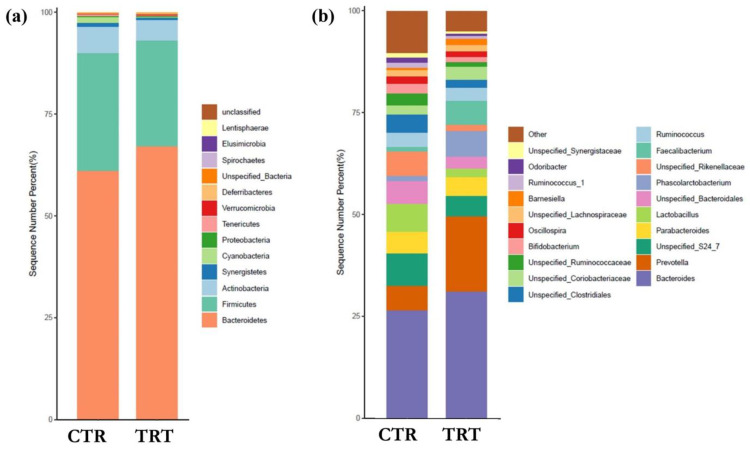
Taxonomic composition of the broiler caecal microbiota at phylum (**a**) and genus (**b**) level. CTR: control group; TRT: treated group with *S. cerevisiae* hydrolysate. (n = 6 replicates (subjects) per group).

Analysis of relative abundance profiles did not reveal statistically significant differences between the SCH-supplemented and control groups, both at phylum and genus level (Figure 2a,b). Nonetheless, some notable trends were observed. At the phylum level (Figure 2a), the caecal microbiota of both groups was dominated by Bacteroidetes and Firmicutes, with TRT group showing a higher proportion of Bacteroidetes (67.08% vs. 61.18%) and a lower proportion of Firmicutes (26.10% vs. 28.82%), resulting in a higher Bacteroidetes-to-Firmicutes ratio. At the genus level (Figure 2b), *Prevotella* increased 3.03-fold in the SCH group (18.30% vs. 6.04%), accompanied by a decrease in genera from the Ruminococcaceae family, reflecting the reduction in Firmicutes observed at the phylum level.

## 4. Discussion

The gut represents not only the main site of nutrient absorption, but also a central node in the regulation of immunity and the systemic homeostasis of the organism [29]. In poultry, its functionality is particularly critical, considering the rapidity of growth [29]. In recent years, the focus on nutritional strategies that can positively modulate both animal performance and gut health has led to the increasing use of functional ingredients [3,30,31]. Among these, SCH is increasingly attracting the interest of the scientific community [4]. This compound, obtained from the controlled breakdown of *S. cerevisiae* cell walls, is characterised by important immunomodulatory, trophic and prebiotic components capable of acting in a multifactorial manner on intestinal physiology [3,4,32]. The results obtained in the present study confirm and extend this scenario, showing that supplementation with SCH resulted in positive and interdependent effects on production performance, gut morphology, expression of tight junctions, inflammatory profile and microbial composition of the cecum.

The analysis of zootechnical parameters showed that supplementation of SCH did not result in significant effects in the starter phase nor in the grower phase, while it produced marked and statistically significant improvements in the finisher phase (in terms of BW, ADG and ADFI), as claimed by Li et al. [33] and Wang et al. [34]. This trend suggests a physiological latency of the effect of the supplement, likely related to the gradual maturation of the digestive system, the stabilisation of the gut microbiota and the establishment of full immune function at the enteric level. Precisely, in the first days of life, the intestinal epithelial barrier of broilers is still maturing, characterised by shorter and less differentiated villi, limited production of endogenous digestive enzymes and increased paracellular permeability [35]. In fact, during the first week post-hatch, the gastrointestinal tract of broilers undergoes rapid structural changes: increases in villus height, crypt depth, submucosal thickness, and segment length significantly enhancing nutrient absorption capacity [35,36]. In this context, the uptake and systemic action of bioactive components derived from SCH—such as peptides, β-glucans and mannan-oligosaccharides—are likely to be insufficient to bring about significant effects on production performance [3]. However, by the finisher phase, gut morphology has consolidated, microbial colonisation has stabilised, and metabolic demands are heightened [37,38,39]. This, combined with metabolic demands related to maximum growth, most probably, leads the animal to be physiologically more receptive to the functional action of these compounds. In addition, the increased absorption efficiency at the enterocyte level in the adult phase [35], associated with improved local immune function, could explain the significant increase in ADG observed in the absence of a parallel increase in FCR, which although not statistically significant shows an improving trend.

Over the entire experimental window (1–42 days), the cumulative effects of supplementation are evident: SCH-treated animals show higher ADG and FI than CTR, with a comparable FCR. Most probably, this suggests that the effect of SCH does not lie so much in a reduction in dietary requirements per unit weight produced, but in an increased ability of the animal to sustain higher growth rates, probably due to a better tolerance to metabolic load and a lower incidence of subclinical inflammatory bowel conditions. The absence of adverse effects on mortality confirms the safety of the supplement and the absence of relevant physiological stress.

Integrated analysis of intestinal function data, including measurements of IRI, mucosal morphometric parameters, sIgA levels, gene expression of inflammatory cytokines and tight junction proteins, reveals a systemic and time-dependent effect of dietary supplementation with SCH, most likely due to a coordinated enhancement of intestinal barrier integrity and efficiency [3]. Although IRI showed no significant differences in most intestinal districts, the selective increase observed in jejunum at 28 days in treated subjects suggests a preferential localisation of the trophic effect of SCH. As reported by Toghyani et al. [40], the jejunum is known to be the segment with the highest metabolic activity in terms of absorption of amino acids, peptides and water-soluble vitamins, and its relative expansion could indicate an increase in the functional share of the intestinal tract induced by the presence of bioactive components in the supplement, such as nucleotides, soluble peptides and polysaccharides derived from the yeast cell wall. These components are known to activate receptor mechanisms at the enterocyte level and to exert an anabolic and immunomodulatory action on the mucosal epithelium [7,17].

The morphometric evidence confirms and deepens this hypothesis: if at 14 days the VH, CD and VCR parameters are comparable between the groups, at 28 days significant changes emerge, with a marked increase in VCR in the jejunum and an even more evident remodelling in the ileum, where VH is increased, CD is reduced and VCR is consequently elevated. At 42 days, this picture is further consolidated, with significantly higher VCR in both the jejunum and ileum of treated subjects. It is known that increased VH and VCR, associated with reduced CD, is indicative of a more differentiated intestinal mucosa, with high cell turnover oriented towards absorption rather than uncontrolled proliferation. Indeed, an increased VCR reflects a more mature and functional intestinal mucosa, characterised by taller villi and shallower crypts, which enhances nutrient absorption efficiency. This improved intestinal architecture supports better feed utilisation, contributing to enhanced growth performance and overall health in broilers [16,29,41]. In this context, SCH may have induced a structural stabilisation of the mucosa by stimulating the proliferation of intestinal stem cells in a controlled manner aimed at the functional regeneration of villi, thanks to the exogenous availability of nucleotides and similar growth factors [41].

In parallel, immune assessment showed a selective treatment effect. Although sIgA levels tended to be higher in treated subjects, they were not significantly different, suggesting that SCH does not act by directly stimulating humoral secretory immunity. This appears consistent with the absence of pro-inflammatory stimulation: gene levels of the pro-inflammatory cytokines TNF-α, IL-1β and IL-6 were significantly reduced at 42 days in the SCH group, while IL-1β was significantly decreased already at 28 days. These data confirm an immunomodulatory and anti-inflammatory action of SCH, as suggested by data reported by Gao et al. [24] and Świątkiewicz et al. [42]. The reduction of pro-inflammatory cytokines at the gene level can be interpreted as a consequence, but also as a cofactor, of improved epithelial integrity, as paracellular permeability—when increased—facilitates the passage of endotoxins and antigens that activate local inflammatory responses [14].

In this respect, the over-regulation of junctional proteins is central to understanding the extent of the treatment effect. ZO-1 and Occludin, two key markers of the epithelial barrier [43,44], were significantly increased at 28 and 42 days in treated subjects. ZO-1 is an essential scaffolding protein for tight junction assembly, while Occludin regulates selective paracellular permeability; their increase suggests that SCH promotes the maintenance of cell polarity and epithelial cohesion [3,43,44]. Claudin-1, although not significantly increased, shows a positive trend, suggesting a generalised but uneven enhancement of tight junctions. It is plausible that the increase in ZO-1 and Occludin, observed as early as 28 days, is responsible for the reduction in translocation of bacterial antigens and, consequently, the suppression of the inflammatory response observed at 42 days. Furthermore, the strengthening of the intestinal barrier could improve the selective absorption of nutrients while minimising metabolic losses related to chronic inflammation and oxidative stress [3].

The internal consistency of these data, combined with their temporal progression, allows us to outline a mechanistic model according to which supplementation with SCH initially acts by promoting the morphological differentiation of the intestinal epithelium and the structuring of cell junctions, in a context of controlled trophic activation. Subsequently, thanks to a more stable epithelial microenvironment and a more cohesive barrier, there is a progressive reduction in innate immune stimulation and stabilisation of mucosal inflammatory tone. This dual effect—structural maturation and containment of inflammation—creates the conditions for optimal nutrient absorption and reduced energy expenditure to maintain intestinal homeostasis, plausibly explaining the performance improvements observed in the finisher phase of the production cycle, when metabolic demand is at its highest [35].

Supplementation with SCH resulted in significant effects on the composition of the caecal microbiota in broilers, suggesting a selective and modulating activity by the bioactive components of the supplement. Alpha-diversity analysis showed a significant increase in the Shannon index in TRT group, indicating greater equity and richness of microbial taxa [45]. This increase in diversity is generally considered a positive index of gut ecosystem stability and functionality, as it correlates with increased resilience to perturbations and improved fermentation efficiency [46]. Although the other indices of richness (Chao1, ACE) and dominance (Simpson) did not show statistically significant differences, their increasing trend in the treated group reinforces the hypothesis of a positive impact of supplementation on the structure of the caecal bacterial community. Analysis of beta diversity (PCoA) did not show a statistically significant separation between groups, suggesting that hydrolysate-induced variation mainly affects specific taxa rather than the entire ecological architecture of the community.

Compositionally, the supplementation resulted in a shift at the phylum level, with a relative increase in *Bacteroidetes* and a concomitant decrease in *Firmicutes*. This rebalancing is of particular interest as the *Firmicutes/Bacteroidetes* ratio has often been associated, in monogastrics, with different metabolic capacities: *Bacteroidetes* are generally more efficient in the degradation of complex polysaccharides and in the production of short-chain fatty acids (SCFAs), while an excess of *Firmicutes* may reflect increased proteolytic activity, sometimes associated with putrefactive fermentations [47]. Recent findings further support the functional relevance of this shift in poultry. Fan et al. [48] demonstrated that chicks with a higher abundance of *Bacteroides* showed increased SCFA production and reduced markers of intestinal inflammation, suggesting improved gut health and metabolic efficiency. Similarly, Zhu et al. [49] reported an increase in *Bacteroides* and a decrease in *Firmicutes* which was associated with improved growth performance and beneficial modulation of the caecal microbiota in broilers.

Taxonomic analysis of the caecal microbial community showed that supplementation with SCH led to targeted remodelling of the microbiota, with significant implications not only for digestive function but also for protection against the main enteric pathogens in broiler chickens, such as *Salmonella spp., Escherichia coli* and *Clostridium perfringens* [50].

The increase in *Prevotella*, a bacterium specialised in the degradation of structural carbohydrates and the production of short-chain fatty acids (SCFA) [51], particularly propionate and acetate, can have a dual protective effect. On the one hand, SCFAs lower intestinal pH [51], creating an environment less favourable to the proliferation of Gram-negative pathogens such as *Salmonella* and *E. coli* [52]; on the other hand, propionate and acetate act as modulators of bacterial gene expression, inhibiting the synthesis of virulence factors and reducing the invasive capacity of these pathogens. Furthermore, *Prevotella* is able to compete effectively for the same ecological niches and energy sources, limiting the availability of fermentable substrates that *Salmonella* and *E. coli* use to grow and adhere to the epithelium [51]. Although SCFAs were not measured directly in this study, recent research in poultry has demonstrated a clear association between higher *Prevotella* levels and increased SCFA production. For instance, Sun et al. [53] observed that SCFA levels rise during development in layer chickens in parallel with the maturation of a Prevotella-rich microbiota. Similarly, Liu et al. [54] reported that dietary manipulation to enrich *Prevotella* abundance led to greater SCFA production, improved laying performance, and reduced hepatic lipid accumulation. Furthermore, Kou et al. [51] identified *Prevotella* as a genus significantly correlated with growth performance traits across livestock species, including broilers. These findings align with broader data showing that increased fibre-fermenting bacteria, such as *Prevotella* and Bacteroides, correlate with enhanced SCFA production and reduced gut inflammation, as demonstrated by Fan et al. [48] in chicks with early microbial colonisation patterns. The increased presence of *Prevotella* in our TRT group is therefore consistent with the observed improvements in gut morphology (e.g., increased villus height, higher VCR), barrier integrity (greater expression of ZO-1 and Occludin), and decreased expression of pro-inflammatory cytokines in the jejunum.

At the same time, the reduction in populations belonging to the *Ruminococcaceae* family and, more generally, *Firmicutes* with proteolytic activity, suggests less recourse to putrefactive fermentation of undigested proteins. This aspect is of considerable importance in the prevention of necrotic enteritis, as *Clostridium perfringens* proliferates in conditions of higher pH and in the presence of metabolites derived from protein degradation (e.g., biogenic amines, ammonia) that damage the mucosa and promote bacterial translocation [55,56]. An environment with a prevalence of carbohydrate fermentation and increased SCFA production tends to suppress the germination of *C. perfringens* spores and limit toxin production [57].

These changes in microbial composition are part of a structural and functional improvement in the intestinal barrier, as evidenced by increased expression of ZO-1 and Occludin and a significant reduction in the pro-inflammatory genes TNF-α, IL-1β and IL-6. Strengthening tight junctions reduces paracellular permeability, limiting the passage of endotoxins (e.g., *E. coli* lipopolysaccharide) and clostridial toxins into the lamina propria, thereby preventing the activation of the inflammatory cascade and the development of subclinical enteropathies [58]. Furthermore, a more intact epithelium and a reduced inflammatory microenvironment reduce the likelihood of persistent colonisation by *Salmonella*, which exploits conditions of dysbiosis and inflammation to establish itself in the chicken intestine [59].

Finally, the greater alpha diversity observed (significant increase in the Shannon index) indicates a microbial community that is more resilient to disturbances and less susceptible to sudden imbalances, a condition that represents a natural ecological barrier against the spread of pathogenic strains. A rich and well-balanced microbiota, supported by the presence of beneficial taxa and SCFA producers, is able to maintain a continuous competitive exclusion effect, limiting the possibility that enteric pathogens commonly found in poultry farms will establish themselves and proliferate to clinically relevant levels [45,46].

Overall, the data suggest that supplementation with *S. cerevisiae* hydrolysate not only improves production performance and intestinal integrity, but may also indirectly contribute to reducing the risk of colonisation and development of *Salmonella* spp., *E. coli* and *C. perfringens*, through a combined action of microbiota remodelling, immune modulation and mucosal barrier strengthening.

## 5. Conclusions

Overall, the results obtained showed that dietary supplementation with SCH in broilers improved production performance, with significant effects on final body weight, average daily gain, and average daily feed intake, although no differences were observed in feed conversion ratio. In addition, SCH supplementation enhanced intestinal morphology and epithelial barrier function, while contributing to the modulation of the intestinal inflammatory response. These effects appear to be attributable, at least in part, to an increase in microbial richness and a remodelling of the composition of the caecal microbiota, with particular enrichment of SCFA producing bacteria. While these findings support the potential use of SCH as a nutraceutical additive in poultry production, the study was limited to physiological and microbiological endpoints. Future investigations should incorporate metabolic profiling, proteomics, and other systems-biology approaches to better elucidate the underlying mechanisms of action, evaluate long-term effects, and optimise application strategies under different rearing conditions. Such advances would help define precise inclusion rates and modes of administration, ultimately facilitating the adoption of SCH as a functional feed additive to improve poultry health and productivity.

## Figures and Tables

**Table 1:** The composition and the nutrient levels of the diets.

Ingredients (%)	Starter (1–14 d)	Grower (15–28 d)	Finisher (29–42 d)
Wheat	64.87	66.26	68.15
Soybean meal (46 CP)	16.04	12.10	8.65
Corn gluten meal (60 CP)	4.00	4.50	5.00
Cottonseed meal	3.00	3.00	3.00
Rapeseed meal	2.00	2.50	2.85
Wheat middlings	2.00	2.00	2.50
Soybean oil	3.45	5.50	5.90
Dicalcium phosphate	1.50	1.25	0.98
Limestone	1.35	1.22	1.28
Salt	0.20	0.18	0.17
DL-Methionine	0.23	0.17	0.16
L-lysine HCl	0.62	0.58	0.60
L-threonine	0.16	0.13	0.13
Vitamin Premix ^1^	0.02	0.02	0.02
Mineral Premix ^2^	0.20	0.20	0.20
Choline chloride (50%)	0.10	0.10	0.10
Sodium bicarbonate	0.20	0.23	0.25
Phytase	0.01	0.01	0.01
NSP enzymes	0.05	0.05	0.05
Total	100.00	100.00	100.00
**Calculated Nutrient levels (%)**			
AME (MJ/kg)	12.35	12.97	13.18
Crude protein	22.00	21.00	20.00
Calcium	1.00	0.90	0.85
Available phosphorus	0.40	0.35	0.30
Lysine	1.25	1.10	1.05
Methionine	0.57	0.50	0.48
Methionine + cystine	0.90	0.81	0.80
Threonine	0.81	0.72	0.68
Tryptophan	0.24	0.21	0.20

CP: Crude Protein; NSP: Non-starch polysaccharides; AME: Apparent Metabolizable Energy. ^1^ The vitamin premix supplied the following per kg of complete feed: vitamin A, 12 500 IU; vitamin D3, 2500 IU; vitamin K3, 2.65 mg; vitamin B1, 2 mg; vitamin B2, 6 mg; vitamin B12, 0.025 mg; vitamin E, 30 IU; biotin, 0.0325 mg. ^2^ The mineral premix supplied the following per kg of complete feed: Cu, 8 mg; Zn, 75 mg; Fe, 80 mg; Mn, 100 mg; I, 0·35 mg, Selenium, 0. Biotin, 0.0325 mg; folic acid, 1.25 mg; pantothenic acid, 12 mg; niacin, 50 mg.

**Table 2:** Nutrient analyses (%) of experimental diets.

Item	CTR	TRT
Starter phase (1–14 d)		
Moisture	9.65	9.31
Crude protein	23.21	23.18
Crude fat	5.04	4.76
Grower phase (15–28 d)		
Moisture	8.67	8.70
Crude protein	22.18	22.29
Crude fat	6.16	6.38
Finisher phase (29–42 d)		
Moisture	8.00	7.96
Crude protein	21.46	21.37
Crude fat	6.78	6.84

CTR: control group; TRT: treated group with *S. cerevisiae* hydrolysate.

**Table 3:** Primer sequences employed for quantitative RT-PCR analysis.

Name	Sequence 5′-3′	GenBank Number
β-actin	F: 5′ -TTGGTTTGTCAAGCAAGCGG-3′	NM_205518.1
	R: 5′ -CCCCCACATACTGGCACTTT-3′	
TNF-α	F: 5′-TGTGTATGTGCAGCAACCCGTAGT-3′	NM 204267
	R: 5′-GGCATTGCAATTTGGACAGAAGT-3′	
IL-1β	F: 5′-GCTCTACATGTCGTGTGTGATGAG-3′	NM_204524
	R: 5′-TGTCGATGTCCCGCATGA-3′	
IL-6	F: 5′-TCTGTTCGCCTTTCAGACCTA-3′	AJ309540
	R: 5′-GACCACCTCATCGGGATTTAT-3′	
IFN-γ	F: 5′-CTCCCGATGAACGACTTGAG-3′	NM_205149.2
	R: 5′-CTGAGACTGGCTCCTTTTCC-3′	
ZO-1	F: 5′-CTTCAGGTGTTTCTCTTCCTCCTC-3′	XM_413773.4
	R: 5′-CTGTGGTTTCATGGCTGGATC-3′	
Claudin-1	F: 5′-ACAACATCGTGACGGCCCA-3′	NM_001013511.2
	R: 5′-CCCGTCACAGCAACAAACAC-3′	
Occludin	F: 5′-GCAGATGTCCAGCGGTTACTAC-3′	NM_205128.1
	R: 5′-CGAAGAAGCAGATGAGGCAGAG-3′	

**Table 4:** Growth performance of the experimental groups (n = 6 replicates (cages) per group).

Item	CTR	TRT	*p*-Value
Starter phase (1–14 d)			
BW at d 1 (g)	41.35 ± 0.37	41.28 ± 0.33	0.938
BW at d 14 (g)	469.9 ± 4.7	471.3 ± 1.7	0.849
ADG (g/d)	28.8 ± 0.4	28.9 ± 1.1	0.846
ADFI (g/d)	35.9 ± 2.17	35.9 ± 1.49	0.995
FCR	1.25 ± 0.05	1.24 ± 0.04	0.921
Grower phase (15–28 d)			
BW at 28 d (g)	1384.1 ± 30.5	1412.2 ± 26.6	0.120
ADG (g)	65.0 ± 2.0	67.1 ± 2.2	0.121
ADFI (g)	106.5 ± 3.6	109.3 ± 3.6	0.213
FCR	1.64 ± 0.04	1.63 ± 0.044	0.743
Finisher phase (29–42 d)			
BW at 42 d (g)	2558.0 ± 64.0 ^b^	2735.1 ± 151.0 ^a^	0.025
ADG (g)	85.8 ± 6.6 ^b^	97.7 ± 11.3 ^a^	0.049
ADFI (g)	161.0 ± 4.0 ^b^	176.4 ± 14.0 ^a^	0.027
FCR	1.89 ± 0.14	1.82 ± 0.16	0.438
Whole phase (1–42 d)			
ADG (g)	57.9 ± 1.7 ^b^	62.7 ± 3.6 ^a^	0.015
ADFI (g)	97.3 ± 1.6 ^b^	103.6 ± 6.1 ^a^	0.034
FCR	1.68 ± 0.06	1.66 ± 0.07	0.509
Mortality (%)	3.53 ± 3.14	5.34 ± 1.98	0.261

CTR: control group; TRT: treated group with *S. cerevisiae* hydrolysate. BW: Body Weight; ADG: Average Daily Gain; ADFI: Average Daily Feed Intake; FCR: Feed Conversion Ratio. Results are expressed as mean ± standard deviation (mean ± SD). Different superscript letters indicate statistically significant differences between the groups (*p* < 0.05).

**Table 5:** Intestine Relative Index (IRI, %) at 14, 28, and 42 d of age (n = 6 replicates (subjects) per group per timepoint).

Ingredients	CTR	TRT	*p*-Value
14 d			
Duodenum	1.64 ± 0.18	1.54 ± 0.15	0.242
Jejunum	2.62 ± 0.23	2.57 ± 0.46	0.791
Ileum	1.86 ± 0.49	1.88 ± 0.31	0.828
28 d			
Duodenum	1.11 ± 0.19	1.20 ± 0.32	0.723
Jejunum	2.18 ± 0.73 ^b^	3.00 ± 0.43 ^a^	0.021
Ileum	1.62 ± 0.46	2.15 ± 0.52	0.136
42 d			
Duodenum	0.71 ± 0.24	0.97 ± 0.23	0.789
Jejunum	1.17 ± 0.33	1.29 ± 0.32	0.424
Ileum	0.98 ± 0.41	0.87 ± 0.13	0.579

CTR: control group; TRT: treated group with *S. cerevisiae* hydrolysate. Results are expressed as mean ± standard deviation (mean ± SD). Different superscript letters indicate statistically significant differences between the groups (*p* < 0.05).

**Table 6:** Intestinal morphology at 14, 28, and 42 days of age (n = 6 replicates (subjects) per group per timepoint).

		CTR	TRT	*p*-Value
14 d				
Duodenum	VH	1227.08 ± 187.76	1278.13 ± 194.07	0.653
	CD	167.21 ± 28.33	162.41 ± 35.75	0.802
	VCR	7.37 ± 0.56	8.02 ± 1.17	0.246
Jejunum	VH	1033.38 ± 117.22	1108.01 ± 209.36	0.473
	CD	150.42 ± 20.85	147.58 ± 17.24	0.814
	VCR	6.91 ± 0.54	7.50 ± 0.99	0.240
Ileum	VH	598.20 ± 41.71	636.75 ± 38.57	0.127
	CD	155.67 ± 33.98	131.95 ± 15.26	0.150
	VCR	4.03 ± 1.05	4.87 ± 0.59	0.118
28 d				
Duodenum	VH	1545.68 ± 359.31	1705.38 ± 198.64	0.725
	CD	199.47 ± 41.06	180.27 ± 26.59	0.707
	VCR	8.08 ± 0.54	8.86 ± 0.92	0.155
Jejunum	VH	1160.73 ± 126.08	1255.54 ± 147.01	0.278
	CD	167.09 ± 26.58	152.15 ± 30.43	0.407
	VCR	7.12 ± 0.75 ^b^	8.39 ± 1.08 ^a^	0.035
Ileum	VH	657.37 ± 49.31 ^b^	729.923 ± 32.93 ^a^	0.013
	CD	151.42 ± 17.25 ^a^	129.97 ± 12.06 ^b^	0.032
	VCR	4.35 ± 0.22 ^b^	5.66 ± 0.73 ^a^	0.002
42 d				
Duodenum	VH	1642.59 ± 237.63	1690.46 ± 327.01	0.797
	CD	186.94 ± 35.04	176.52 ± 22.02	0.592
	VCR	8.86 ± 0.59	9.51 ± 0.69	0.152
Jejunum	VH	1248.41 ± 180.71	1471.71 ± 209.60	0.076
	CD	179.60 ± 20.76	154.79 ± 32.69	0.148
	VCR	6.96 ± 0.74 ^b^	9.65 ± 0.91 ^a^	<0.001
Ileum	VH	939.14 ± 93.06	1076.47 ± 139.32	0.073
	CD	158.05 ± 16.66	139.91 ± 21.71	0.136
	VCR	5.96 ± 0.49 ^b^	7.73 ± 0.62 ^a^	<0.001

CTR: control group; TRT: treated group with *S. cerevisiae* hydrolysate. VH: Villi height, CD: crypt depth and VCR: villus/crypt ratio. Results are expressed as mean ± standard deviation (mean ± SD). Different superscript letters indicate statistically significant differences between the groups (*p* < 0.05).

**Table 7:** Intestinal secretory IgA (sIgA) at 28 and 42 days (n = 6 replicates (subjects) per group per timepoint).

	CTR	TRT	*p*-Value
28 d			
Jejunum	318.25 ± 58.33	336.99 ± 52.52	0.571
Ileum	201.59 ± 24.70	220.33 ± 34.95	0.309
42 d			
Jejunum	334.32 ± 40.91	353.66 ± 43.64	0.461
Ileum	251.59 ± 55.97	270.33 ± 40.24	0.521

CTR: control group; TRT: treated group with *S. cerevisiae* hydrolysate. Results are expressed as mean ± standard deviation (mean ± SD).

**Table 8:** Gene expression analysis of inflammatory cytokines in the jejunum of broilers at 28 and 42 days (n = 6 replicates (subjects) per group per timepoint).

	CTR	TRT	*p*-Value
28 d			
TNF-α	1.09 ± 0.19	0.89 ± 0.29	0.392
IL-1β	1.06 ± 0.26 ^a^	0.73 ± 0.18 ^b^	0.018
IL-6	1.00 ± 0.37	0.93 ± 0.26	0.866
IFN-γ	1.03 ± 0.34	0.80 ± 0.23	0.346
42 d			
TNF-α	1.00 ± 0.08 ^a^	0.65 ± 0.20 ^b^	<0.001
IL-1β	1.02 ± 0.24 ^a^	0.67 ± 0.15 ^b^	0.002
IL-6	1.04 ± 0.18 ^a^	0.78 ± 0.20 ^b^	0.049
IFN-γ	1.09 ± 0.28	0.79 ± 0.22	0.158

CTR: control group; TRT: treated group with *S. cerevisiae* hydrolysate. TNF-α: Tumour Necrosis Factor alpha, IL-1β: Interleukin 1 beta, IL-6: Interleukin 6, IFN-γ: Interferon gamma. Results are expressed as mean ± standard deviation (mean ± SD). Different superscript letters indicate statistically significant differences between the groups *(p <* 0.05).

**Table 9:** Gene expression levels of the tight junction proteins Zonula Occludens-1 (ZO-1), Occludin, and Claudin-1 in the jejunum of broiler chickens at 28 and 42 days (n = 6 replicates (subjects) per group per timepoint).

	CTR	TRT	*p*-Value
28 d			
ZO-1	1.07 ± 0.32 ^b^	1.87 ± 0.63 ^a^	<0.001
Occludin	1.01 ± 0.38 ^b^	1.89 ± 0.80 ^a^	0.005
Claudin-1	1.01 ± 0.29	1.38 ± 0.51	0.454
42 d			
ZO-1	1.01 ± 0.30 ^b^	2.86 ± 0.89 ^a^	<0.001
Occludin	1.06 ± 0.25 ^b^	2.52 ± 0.63 ^a^	<0.001
Claudin-1	1.07 ± 0.41	1.66 ± 0.74	0.108

CTR: control group; TRT: treated group with *S. cerevisiae* hydrolysate. ZO-1: Zonula Occludens-1. Different superscript letters indicate statistically significant differences between the groups (*p* < 0.05). Results are expressed as mean ± standard deviation (mean ± SD).

## Data Availability

The data underlying this article will be shared on reasonable request to the corresponding author.
